# Role of *IL-6*, *IL-10* and *TNFα* Gene Variants in Preterm Birth

**DOI:** 10.3390/jcm13082429

**Published:** 2024-04-21

**Authors:** Mirta Kadivnik, Deni Plečko, Kristina Kralik, Nena Arvaj, Jasenka Wagner

**Affiliations:** 1 Clinic of Obstetrics and Gynecology, University Hospital Center Osijek, J. Huttlera 4, 31000 Osijek, Croatia; mirta.kadivnik@gmail.com; 2 Department of Obstetrics and Gynecology, Faculty of Medicine, J.J. Strossmayer University, J. Huttlera 4, 31000 Osijek, Croatia; 3 Department of Medical Biology and Genetics, Faculty of Medicine, J.J. Strossmayer University, J. Huttlera 4, 31000 Osijek, Croatia; deni.plecko@gmail.com (D.P.);; 4 Department of Medical Statistics and Informatics, Faculty of Medicine, J.J. Strossmayer University, J. Huttlera 4, 31000 Osijek, Croatia

**Keywords:** premature birth, single nucleotide polymorphisms, inflammation, cytokines, interleukins

## Abstract

**Background:** The association of gene variants for *interleukin 6 (IL-6)* (rs1800796), *interleukin 10 (IL-10)* (rs1800896) and *tumor necrosis factorα (TNFα* (rs1800629) with the occurrence of spontaneous preterm birth (PTB) was investigated to determine whether these genetic variants are a risk factor. **Methods:** A total of 199 blood samples from pregnant women who had given birth prematurely and 200 control blood samples were analyzed to determine single nucleotide polymorphisms (SNPs) of genes for *IL-6* (rs1800796), *IL-10* (rs1800896) and *TNFα* (rs1800629). The control samples were samples from pregnant women with term delivery. The isolation of DNA was performed on mini-spin columns according to the manufacturer’s protocol. The quality and purity of the isolated DNA were tested using a Qubit 3 fluorometer. Genotyping was performed with an ABI PRISM 7500 SDS using TaqMan SNP genotyping assays. The genotypes obtained were analyzed using the 7500 Software v2.3 package. **Results:** Carriers of the A/A genotype for the rs1800629 SNP of the *TNFα* gene have a 4.81 times greater chance of late-onset PTB compared to carriers of the G/G and A/G genotypes in the recessive inheritance model. The presence of the G/G genotype in the recessive inheritance model compared with the G/A and A/A genotypes for the rs1800896 SNP of the *IL-10* gene represents a potentially protective factor, with mothers in the term-birth group having an almost 2-fold lower odds of PTB in general and an almost 10-fold lower odds of early PTB. On the other hand, carriers of the A/G genotype of rs1800896 have a 1.54-fold higher chance of preterm birth in general and a 1.6-fold higher chance of late preterm birth in the superdominant inheritance model compared to the A/A and G/G genotypes in the group of mothers with PTB. In this study, no association was found between PTB and the rs1800796 SNP of the *IL-6* gene. **Conclusions:** rs1800629 in mothers was associated with PTB. rs1800896 shows a potentially protective effect for the occurrence of PTB in this study. No association was found between PTB and rs1800796.

## 1. Introduction

Premature birth (PTB) is defined by the World Health Organisation (WHO) as a live birth before 37 weeks of gestation [1,2]. The percentage of PTBs in the world in 2020 was 9.9%, going from about 6.8% in eastern Asia, south-eastern Asia and Oceania to 13.2% in southern Asia (even 16.2% in Bangladesh) [3]. In the Republic of Croatia, the percentage of PTB ranged from 6.19% to 6.97% between 1994 and 2014 [4]. According to studies, preterm birth is associated with 70% of neonatal mortality and 75% of neonatal morbidity [5].

A recognized risk factor for PTB is maternal and/or fetal genetic predisposition, confirmed in many epidemiological studies [6,7]. Studies have shown that the risk of PTB is higher for women born prematurely, for mothers who have had a previous PTB and for mothers whose sisters, mothers, or female cousins have had a PTB [8].

One of the potential underlying molecular mechanisms of PTB is an imbalance between anti-inflammatory and pro-inflammatory metabolic pathways [9]. During pregnancy, the transition from a quiescent to a pro-inflammatory environment triggers labor and involves a three-stage process characterized by uterine contractility, cervical ripening and rupture of the membranes [10]. Infection, stress and obesity are all known to promote inflammation [11], which suggests that these environmental exposures may promote an inflammation-mediated mechanism resulting in early parturition. Cytokines are critical to the initiation and regulation of this process [9].

Labor begins with an increase in interleukin (IL) gene expression, which is promoted by leukocytes, infiltrating the myometrium and cervix [12]. Increases in interleukin 1-beta (IL1β) and tumor necrosis factor α (TNFα) stimulate myometrial contractions by increasing calcium entry into myometrial smooth muscle cells [13]. Cervical ripening is an inflammatory event in both term and preterm labor [14]. Pro-inflammatory cytokines then stimulate the expression of other inflammatory modulators, such as matrix metalloproteinases (MMPs), which promote extracellular matrix degradation and cervical remodeling [15]. In some women, however, infectious agents are not identified despite high levels of pro-inflammatory cytokines. In that case, these high levels are presumably caused by other factors, e.g., a genetically determined predisposition to an upregulated synthesis of pro-inflammatory cytokines related to the polymorphisms of the corresponding genes [16].

There is much information from candidate gene studies on variants of inflammatory genes associated with an increased risk of PTB. Most single nucleotide polymorphisms (SNP) of cytokine genes are located in the regulatory regions of the gene and directly affect its transcriptional activity and the concentration of the cytokine in the blood [17]. Since certain genes regulate the corresponding cytokines, maternal genetic polymorphisms have been studied to evaluate their association with PTB [18,19]. Most studies have analyzed SNPs in genes encoding *TNF α*, *interferon γ* (*IFNγ*), *interleukin 6 (IL-6)* and *interleukin 10 (IL-10)* [20,21,22,23,24,25,26,27,28,29,30,31,32,33,34,35,36,37,38,39].

A highly studied gene in PTB is the *TNFα* gene. It is located on chromosome 6 and consists of 2772 base pairs [40]. It encodes a multifunctional pro-inflammatory cytokine that belongs to the tumor necrosis factor (TNF) superfamily. Its most common polymorphism is located in the promoter region (C->A; rs1800629) and is associated with reduced circulating TNFα levels. The mutant allele (A) is associated with higher TNFα expression, which leads to an upregulation of the inflammatory pathway and is associated with PTB risk [41,42].

Another pro-inflammatory cytokine that is frequently studied is *IL-6*. It is located on chromosome 7 and consists of six exons and five introns. It has 6119 base pairs. This gene encodes a cytokine that plays a role in inflammation and the maturation of B cells. In addition, the encoded protein is an endogenous pyrogen that can trigger fever in people with autoimmune diseases or infections [40].

A polymorphic site in the *IL-6* gene at position −572 (G->C; rs1800796) is located in an intron that regulates transcription and is associated with reduced promoter activity of the mentioned gene. The C/C variant leads to reduced IL-6 production, while heterozygous G/C or homozygous G/G variants show normal production of IL-6 [28].

In contrast to the increased production of pro-inflammatory cytokines associated with PTB, other cytokines (e.g., the anti-inflammatory cytokine IL-10) may exert a counteracting protective effect on pregnancy. The suppression of the pro-inflammatory arm ensures that the pregnancy lasts until the end. IL-10 deactivates macrophages and causes the synthesis of TNFα, IL-6, IL-1 and IL-8 to be silenced, thereby interrupting the inflammatory pathway and limiting inflammation-related tissue pathology and the cascade of other pro-inflammatory cytokines and chemokines [43]. An in vitro study has shown that IL-10 plays a critical role in downregulating pro-inflammatory cytokines and progesterone in the fetal amniochorionic membranes, limiting negative inflammatory responses [43].

The *IL-10* gene is located on chromosome 1 and consists of 6940 base pairs [40]. It is polymorphic, leading to quantitative differences in cytokine levels achieved in different individuals [44]. The most common variant of the *IL-10* gene is a single nucleotide substitution of adenine for guanine at position −1082 (rs1800896). IL-10 production was highest in individuals homozygous positive for the guanine allele (G/G) [45]. The IL-10-1082 GG and GA genotypes may be related to the level of IL-10 expression [44].

Based on the previous studies, our study aimed to evaluate the role of three selected genetic variations in the maternal *TNFα, IL-6* and *IL-10* genes and to identify women who may be at higher or lower risk for PTB compared to the general population. These genetic markers can also be used for the risk stratification of pregnancies.

## 2. Materials and Methods

### 2.1. Study Subjects

This case-control study was conducted between November 2017 and March 2024 at the Department of Gynecology and Obstetrics at Osijek University Hospital Center and at the Medical Genetics laboratory at the Department of Medical Biology and Genetics, Faculty of Medicine, Osijek. Relevant anamnestic and sociodemographic information was collected in cooperation with all participants included in the study.

Two groups of pregnant women participated in the study (200 women who had given birth at term and 199 women who had PTB). None of the mothers in either group were genetically related.

The inclusion criterion for pregnant women in the premature labor group was the birth of a single live newborn before 37 weeks of gestation with a spontaneous onset of labor with or without a preterm premature rupture of membranes (PPROM) and hospital admission after the onset of labor.

The exclusion criteria for the premature labor group were already known risk factors for preterm birth (such as in vitro fertilization, multiple pregnancies, any cervical surgery, inflammation of the reproductive organs and kidney disease) as well as pregnancy complications (gestational diabetes mellitus, hypertension in pregnancy) or signs of infection in laboratory tests shortly before birth or signs of infection in the pathohistological analysis of the placenta. In addition, all pregnant women with a positive personal or family history of preterm birth were excluded from the study.

The group of pregnant women with premature birth was, for further analysis, sub-divided into three subgroups by gestational age: extremely early PTB group (24–27 + 6 weeks of gestation, *n* = 16), early PTB group (28–31 + 6 weeks of gestation, *n* = 32), and late PTB group (32–36 + 6 weeks of gestation, *n* = 151) [3].

The control group included healthy women who delivered single newborns between 37 and 41 + 3 weeks of gestation.

If the pregnancy was uncomplicated, all deliveries ended naturally. Control subjects were matched for age, socioeconomic and demographic status, ethnicity, prenatal care and mode of delivery.

The gestational age of all subjects was determined according to the first day of the last menstrual cycle and confirmed by ultrasound findings in the first trimester. In the case of a mismatch between the due date concerning the first day of the last menstrual cycle and the ultrasound finding, a correction of gestational age was made according to the ultrasound finding [46].

Sociodemographic, epidemiological and clinical data were collected in cooperation with the mothers. Available medical documentation on the pregnancy and childbirth of the mothers was used. Data related to the physical status of pregnant women during pregnancy, family and personal history, habits and previous events in pregnancy were collected.

### 2.2. Blood Sampling and Analysis

The venous blood of pregnant women was collected only once after obtaining informed consent. Three described genetic variants of genes *IL-6* (rs1800769), *IL-10* (rs1800869) and *TNFα* (rs1800629) were analyzed. Variants were selected based on previously published associations with PTB [20,25,26,30,32,36,47,48] and are listed along with known functions in Table 1.

Blood was taken from the mothers after admission to the delivery room, in the first stage of labor. For the analysis of genetic variants, a total of 3 mL of the mother’s venous blood was collected in vacuum tubes (Vacutainer, Becton Dickinson, Franklin Lakes, NJ, USA) with anticoagulant ethylenediaminetetraacetic acid (EDTA).

Genomic deoxyribonucleotide acid (DNA) was extracted from 200 µL of EDTA anticoagulated whole blood using commercially available spin colons for DNA extraction QIAamp DNA Blood Mini Kit (Qiagen GmBH, Hilden, Germany) according to the manufacturer’s instructions [61]. DNA samples were stored at −20 °C until further analysis. The quality and purity of the isolated DNA were tested using a Qubit 3 fluorometer.

SNP genotype analysis was performed using TaqMan-based fluorescent probes (TaqMan SNP Genotyping Assays, Waltham, MA, USA) on the ABI PRISM 7500 real-time polymerase chain reaction (PCR) system (Applied Biosystems, Foster City, CA, USA) [62]. The thermocycling procedure consisted of the following: 1 hold at 95 °C for 10 min; 40 cycles of denaturation at 92 °C for 15 s and primer annealing and extension at 60 °C for 1 min. Negative and positive control samples were run simultaneously within each analyzed real-time PCR plate. The total reaction volume per well was 25 µL with 2 µL of DNA used as a template. The allelic discrimination analysis was performed using SDS 7500 Software Version 2.3 (Applied Biosystems, Foster City, CA, USA).

### 2.3. Statistical Analysis

Analyses were performed using the SNPStats web tool (Solé et al., 2006) [63], SHEsisPlus [64,65], MedCalc^®^ Statistical Software version 19.6 (MedCalc Software Ltd., Ostend, Belgium; https://www.medcalc.org; accessed on 1 March 2020) and SPSS (IBM Corp. Released 2015. IBM SPSS Statistics for Windows, Version 23.0. IBM Corp., Armonk, NY, USA).

Absolute and relative frequencies represented categorical data. The median and interquartile range described continuous data in cases of deviation from normal distribution. The variance of the categorical variables was tested using the Chi-squared test and Fisher’s exact test. The Mann–Whitney U test was employed to test differences between variables in two independent groups. To further assess the presence of associations, we calculated the odds ratios (OR) and their respective 95% confidence intervals (CI). The impact of multiple factors on the probability of preterm birth was assessed through logistic regression (*backward method*). An additional level of genotyping quality control was performed using the Chi-Square goodness of fit test, by comparing our genotype distribution with those predicted by the Hardy–Weinberg equilibrium.

Genotype analysis and haplotype analysis (with the correction of *p*-values using the Benjamini–Hochberg method) were performed using the online programs SNPstats and SHEsisPlus. Bonferroni correction was applied for all multiple analyses. The Hardy–Weinberg equilibrium of genotypic frequencies was tested using the χ^2^ test with degrees of freedom (df = 1) through the SHEsis Plus online program. The D’ coefficient was employed to describe linkage disequilibrium (LD).

All *p*-values are two-tailed. The significance level was set at Alpha = 0.05.

## 3. Results

### 3.1. Statistical Analysis of Demographic Characteristics of Cases and Controls

For this case-control study, 399 pregnant mothers were included. Mothers were divided into groups of mothers with term (*n* = 200) and premature (*n* = 199) birth, based on their gestational age at delivery. The demographic characteristics of mothers and infants in both groups and selected risk factors for PTB are listed in Table 2.

### 3.2. Statistical Analysis of Genotype and Allele Distribution of Three SNPs of Genes IL-10, IL-6 and TNFα in Various Inheritance Models between Cases and Controls

The genotype frequencies of all three SNPs studied in both the study and control groups were in Hardy–Weinberg equilibrium (*p* > 0.05).

There was no statistically significant difference between the group of mothers with term and PTB in the distribution of genotype and allele frequencies of all three selected SNPs.

On the other hand, there was a statistically significant difference in the genotype distribution of the G/G genotype of the rs1800896 SNP of the *IL-10* gene in the recessive inheritance model between the group of mothers with term birth and PTB. Mothers with the G/G genotype of the abovementioned gene have almost two times less chance of having PTB (*p* < 0.04, chi-square test). In addition, there is a statistically significant difference in the distribution of heterozygotes A/G in the superdominant model of inheritance of the rs1800896 SNP of the *IL-10* gene. Mothers with this genotype are 1.5 times more likely to have PTB (*p* < 0.03, chi-square test) (Table 3).

There were no statistically significant differences in the genotype distribution and allele frequencies of the three studied SNPs between the three subgroups of mothers with PTB (Table 4).

There was no statistically significant difference between the two groups of mothers with term birth and extremely early PTB in the distribution of the genotype and allele frequencies of all three selected SNPs (Table 5). There was also no statistically significant difference in the genotype distribution between these two groups according to different inheritance models (Table 5).

When we compared the subgroup of mothers with early PTB and a group of mothers with term birth, we found a statistically significant difference in the distribution of the GG genotype of the rs1800896 SNP of the *IL-10* gene between these two groups. Mothers with the GG genotype in the recessive model of inheritance have around 10 times lower chance for PTB (*p* < 0.03, Chi-squared test) (Table 6).

When we compared a subgroup of mothers with late PTB and a group of mothers with term birth, we found statistically significant differences in the genotype distribution of two of the studied SNPs. In the superdominant model of inheritance, mothers who have the A/G genotype of the rs1800869 SNP of the *IL-10* gene have 1.60 times more chance of having late premature birth (*p* < 0.03, chi-square test) (Table 7). It is also relevant to mention that there is an almost statistically significant difference in the genotype distribution of the rs1800629 SNP of the *TNFα* gene. Namely, mothers who have the GG genotype of the mentioned SNP in the recessive model of inheritance, have 4.81 times more chance for late premature birth (*p* < 0.03, chi-square test) (Table 7).

### 3.3. Statistical Analysis of Linkage Disequilibrium (LD) and Haplotypes Analysis of Three SNPs of Genes IL-10, IL-6 and TNFα

Next, we investigated the possible association between a combination of different pro- and anti-inflammatory genotypes (haplotypes) and pregnancy outcomes. There were no statistically significant differences in the frequencies of the individual haplotypes between the two groups studied (Table 8).

**Figure 1:**
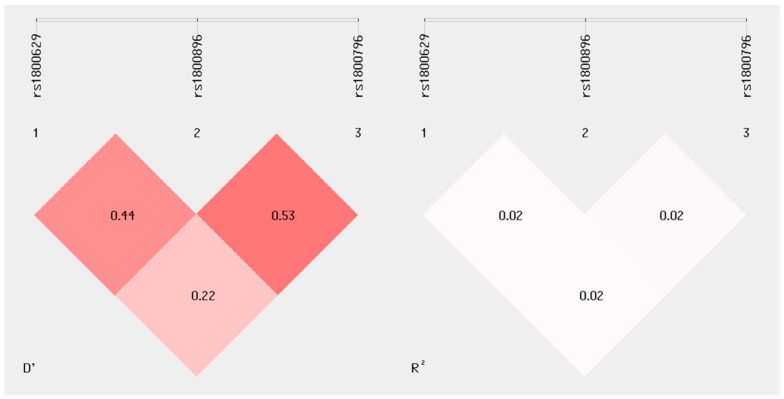
Example analysis of allelic linkage disequilibrium (LD) of three selected SNPs (*IL-10*, *IL-6* and *TNFα*) in the group of mothers. The calculations in the SHEsis plus program show the three selected SNPs of the genes *IL-10*, *IL-6* and *TNFα* in the upper part. The lower part of the image shows the values of the allelic linkage disequilibrium (LD) for pairs of the specified polymorphism values of r2 and D’. Both values are standardized values for LD. If the values for the correlation coefficients r^2^ and D’ are closer to zero, the population is closer to equilibrium. The highest allelic linkage disequilibrium was observed between SNPs rs1800896 and rs1800876 concerning D’ values.

Linkage disequilibrium (LD) between three chosen SNPs of genes *IL-10*, *IL-6* and *TNFα* in a group of mothers is shown in Figure 1. The greatest LD is seen between rs 1800796 and rs1800896 (D’ 0.53).

### 3.4. Prediction of Probability for PTB by Bivariate and Multivariate Logistic Regression Analysis

Bivariate and multivariate logistic regression was performed to predict the probability of PTB for the three SNPs mentioned, with correction for risk factors (smoking habit, maternal age, maternal body mass index, newborn gender and bleeding during pregnancy) as possible predictors of PTB. The multivariate logistic regression (backward), results in a significant model for the prediction of SPP, in which the mother’s rs1800629 (A/G-A/A vs. G/G) is significant (*p* = 0.02, OR = 2.09, 95% CI 1.13–3.88)). The model is fully significant (χ^2^ test = 30.7, df = 6 *p* < 0.001) and explains between 7% (according to Cox and Snell) and 10% (according to Negelkerke) of the occurrence of sPP and correctly classifies 61.8% of the cases (Table 9).

## 4. Discussion

Nowadays, cytokine gene polymorphisms are extensively studied, and the role of pro- and anti-inflammatory cytokines in developing PTB is now recognized. We investigated three gene variants in different genes that regulate immunity and the balance of pro- and anti-inflammatory cytokines. The study was conducted to gain a better understanding of the genetic predisposition to PTB in the European population. As far as we could find in the literature, this was the first study that included these three SNPs of the genes for *Il-10*, *IL-6* and *TNFα* in the Croatian population.

We examined two SNPs affecting the expression of genes encoding pro-inflammatory cytokines, *IL-6* and *TNFα*, and one SNP which influences the expression of the gene coding anti-inflammatory cytokine, *IL-10*. We have confirmed the association of two SNPs with a higher or lower risk in mothers for PTB.

When evaluating the association of the rs1800896 SNP of the *IL-10* gene with PTB, we found a significant association with PTB in mothers who had the GG or AG genotype. The GG genotype was found to be protective for PTB. Carriers of this genotype had a 10-fold lower risk of early PTB and a 2-fold lower risk of PTB in general (both in the recessive inheritance model). In contrast, carriers of the AG genotype had a 1.5-fold higher risk of PTB overall and a 1.6-fold higher chance for late-onset PTB (both in the superdominant model of inheritance).

Several studies have investigated the association between the rs1800896 SNP of the *IL-10* gene and PTB. Some of them supported our results. Pandey et al. found an association of the rs1800896 SNP with PTB. They found that mothers with the GG genotype were 2-fold less likely to have PTB in an additive or dominant model of inheritance for this SNP [26]. They have also found that carriers of the A allele of this SNP in the haplotype with two other alleles have a higher possibility for PTB. Lybomirskaya et al., showed in their study of 50 women with PTB between 24–32 weeks of gestation that the rs1800896 SNP in the haplotype with four other SNPs (rs2243250 of *IL-4*, rs4742076 and rs3758239 of *relaxin 2* (*RLN2)* and rs1800872 of *IL-10*) was significantly associated with PTB [25]. Finally, Menon et al. showed in their study the association of rs1800896 with the IL-10 concentration in the amniotic fluid (AF) of Caucasian pregnant women, which is indirectly related to the possibility of PTB [21]. The GG genotype of the aforementioned SNP increases the IL-10 level in AF.

In contrast to these results, four other studies found no association of this SNP of IL-10 with PTB [27,28,38,66].

Il-10 is an anti-inflammatory cytokine that suppresses the expression of inflammatory cytokines such as TNFα and IL-6. Clinical studies showed that lower IL-10 levels were significantly associated with PTB and may be biomarkers for PTB. Studies also showed that the protective effect of the GG genotype of rs1800896 could be due to the increase in IL-10 serum level [55].

Genetic variations can influence the expression of genes and serve as genetic markers for disease susceptibility or severity. In our study, the GG and GA genotypes were found to have opposite effects on PTB possibility, meaning that carriers of the GG genotype have a lower probability of PTB, but carriers of the GA heterozygote genotype have a higher probability of PTB. As we have shown, our results were supported by the findings of three other authors. The possible reason for the inconsistencies with the results of the other four studies could be related to the characteristics of the patients enrolled in each study, ethnic differences between the study populations and different sample sizes of the studies. As Turner et al. have shown, this SNP is located in the Ets binding site of the *IL-10* gene, so these genotypes may be related to the level of IL-10 expression [44].

Based on all of this, our results confirm the protective role of IL-10 for PTB and the influence of rs1800896 on the prediction of PTB.

When evaluating the association of rs1800629 of the *TNFα* gene with PTB, a significant association with PTB was found in the maternal data in mothers who had the AA genotype. In the recessive inheritance model, mothers who had the AA genotype were 4.8 times more likely to have late-onset PTB. Importantly, after applying bivariate and multivariate logistic regression, TNFα-308 was also the only significant predictor with a more than 2-fold higher risk of PTB in carriers of the GA heterozygote genotype.

There are several findings on the *TNFα*-308G/A genotype and PTB that support our findings. In a study by Chen et al. on familial triads (mother, father and newborn), the association of *TNFα*-308 A with PTB was established [51]. In their study, Yilmaz et al. showed that a combination of maternal and fetal genotypes indicates that the *TNFα*-308 GA genotype is associated with term pregnancy. The maternal *TNFα*-308 GA genotype in combination with a fetal GG genotype leads to a higher risk of PTB [32]. Harper et al., showed in their study of 834 American individuals that mothers with the *TNFα*-308 AA genotype had an almost 2-fold higher risk of PTB [24]. Ramos, Mendes, et al. [39], Drews-Piascek et al. [50] and Moura et al. [42] also showed in their studies that women with the *TNFα*-308 GG genotype in haplotype with other genotypes of SNPs of *TNFα, IL-6* and *IFN γ* have a significant risk of PTB. Drews-Piascek et al. showed that the *TNFα*-238GG/-308GG/-376GG haplotype is protective for PTB and the *TNFα*-238GA/-308GG/-376GG haplotype increases the risk of PTB [50]. Ramos, Mendes, et al. showed a protective role of the *TNFα*-238GG/-308GG haplotype for PTB [39]. Moura et al. showed an association of SNPs with PTB as part of the *TNFα-308*(GG)/*IL6-174*(GG)/*IFN γ +874*(AA) haplotype.

On the other hand, some other studies such as those by Andalas et al. [34], Amory et al. [29], Jafarzadeh et al. [37], Belousova et al. [67], Nuk et al. [27] and Mattar et al. [38] did not report a significant correlation between the *TNFα* -308 A allele and PTB.

TNFα is involved in the remodeling of the cervix and fetal membranes by promoting the production of collagen-degrading matrix metalloproteinases (MMPs), including cervix MMP1 and MMP9 [68]. Several gene polymorphisms within the *TNFα* gene sequence are known. The *TNFα*-308A allele is located in the promoter region of the *TNFα* gene. Individuals who have one or more copies of the *TNFα*-308A allele produce slightly more TNFα than individuals with two copies of the major alleles [69]. The A allele is associated with increased transcriptional activity. Therefore, it is thought that individuals with the TNFα -308 A allele overreact to infections and are more likely to suffer frequent complications from infections [32].

Our study supports the above findings of the association of the rs1800629 SNP of the *TNFα* gene with PTB. The difference between our study and other studies is that we did not investigate fetal and paternal SNPs of *TNFα*-308A, as some of the studies, like the studies by Yilmaz et al. [32] and Chen et al. [51], did. Also, there are different ethnicities in our study and some other studies, which account for a difference between our results and those of Ramon et al. for example. Also, TNFα affects cervical remodeling and the remodeling of amniotic membranes, so there is a possibility that women with PTB and simultaneously, carriers of this SNP could have more PTB associated with preterm premature rupture of amniotic membranes (PPROM). This was shown in the study by Ramon et al. In our study, we did not investigate the association of PTB associated with PPROM with this SNP. This could be the basis for the next study and analysis.

The third SNP we investigated, the rs1800796 SNP of the *IL-6* gene showed no association with PTB.

The SNP rs1800796 in the promoter region of the *IL-6* gene is known to regulate the expression of the IL-6 gene and showed a significant association with unfavorable pregnancy outcomes. IL-6 can trigger the inflammatory response in the acute phase by inducing T lymphocytes, C-reactive protein and B cells differentiation. IL-6 concentrations are elevated in the amniotic fluid, cervical mucus and maternal serum of premature infants [48].

Our results are consistent with several other studies. For example, Sayaril et al. found a low association of the rs1800796 SNP of the *IL6* gene with PTB, although they found a high expression of IL-6 in the blood and placenta of 50 women with PTB [33]. Similarly, no association was found in a study by Ramos et al. [39], Bitner and Kalinka [35] and Sata et al. [70]. Some of the studies confirmed the association of rs1800796 with PTB, but only as part of the haplotype. Han et al. showed that a combination of the genotypes of three SNPs (rs1800796CC-rs1800792CA-rs1800630CA) plays a protective role for PTB [30]. Velez et al. showed that the maternal G-A-G-C haplotype of *IL-6* gene SNPs is strongly associated with PTB [20].

Our results practically confirmed most of the previously mentioned results from other studies. The only association between the *IL-6* gene and PTB was in a case where the rs1800796 was part of the haplotype. Our results and the results of Han et al. [30] and Velez et al. [20] point to the limitations of the association of single loci in a complex phenotype such as PTB. It is possible that the genetic variants we investigated do not influence PTB on their own but could do so in conjunction with other variants or other endogenous or exogenous risk factors.

Regarding the haplotypes, none of the haplotypes of the three SNPs analyzed were significantly associated with PTB in our study. Possibly the highest association, but not significant, could be the *TNFα-308*G-*IL-10*G-*IL-6*G haplotype of SNPs rs1800629-rs1800896-rs1800796 (*p* 0.22, OR 0.83 (95% CI 0.61–1.11), chi-square test) with a possible protective association with PTB.

Interestingly, some studies showed associations of some investigated SNPs with PTB only as part of the haplotype. Moura et al. showed an association of SNPs with PTB as part of the *TNFα-308*(GG)/*IL6-174*(GG)/*IFNG+874*(AA) haplotype [42]. In their study, Han et al. also showed the association of the SNP of the *IL-6* gene rs1800796 with PTB only as part of the haplotype with SNPs of the genes for *IL-10* and *TNFα* (genotypes CC/CA/CA and genotypes CG/CA/CC) [30]. Velez et al. also showed a similar association with PTB for the rs1800796 SNP as part of the haplotype defined by rs12700386–rs1800797–rs1800796–rs1800795 [20].

Our results differ from the results of the studies mentioned. The possible reason for the different results between our study and the mentioned studies could be different combinations of SNPs in the haplotype. In addition, as we have already mentioned, other endogenous or exogenous risk factors and different ethnicities could play a role in other studies.

There are certainly some limitations to our study. First, the number of subjects in the extreme PTB and early PTB subgroups is small. This could be a consequence of the rather low rate of PTB in our country, which is partly due to good perinatal practice and the medical care of pregnant women and partly due to ethnicity (all studied subjects were of Caucasian origin), which is known to have almost the lowest rate of PTB [71]. The proportion of extremely early PTB among all PTBs in the world is approximately 4.2% [3]. Our figure is very similar to the previously mentioned worldwide standard (about 4.2%).

We did not analyze either fetal or paternal DNA. The relative contribution of maternal and fetal genotype to preterm birth is not yet clear, but studies have shown that the fetal genotype also has an impact on PTB. Therefore, this should be included in future research studies.

Furthermore, this is a single-center study. While this design ensures consistency in diagnosis and treatment practices, the choice of hospital by pregnant women may also reflect biases such as social background.

In addition, we examined only one polymorphism for each of the three genes. Preterm birth is widely recognized as a heterogeneous disorder. Therefore, a confirmation of the genetic effects of multiple polymorphisms in *IL*-*6*, *IL*-*10* and *TNFα* is needed in further studies.

PTB is likely to be etiologically heterogeneous. Although we only recorded spontaneous PTB, we could not specify the types of PTB in detail, so a better subdivision of PTB groups based on etiology is recommended in future studies.

The study also has some strengths. The women in this study represent an ethnically homogeneous cohort, which avoids methodological problems with ethnic differences in allele frequencies and genetic admixture. Gestational age was determined using standardized criteria including early pregnancy sonography. Furthermore, in contrast to most of the other studies mentioned, in our study, a group of mothers with PTB was divided into three subgroups and it was found that different subgroups of mothers with PTB showed different associations with the three SNPs investigated. This could lead to possible different pathogenetic pathways of each subgroup of PTB and shed some more light on the pathogenesis of PTB.

## 5. Conclusions

In summary, our results suggest that the rs1800629 SNP of the *TNFα* gene is associated with late-onset PTB. It is shown that mothers who are carriers of the AA genotype of the rs1800629 SNP in the recessive inheritance model have an almost 5-fold higher risk of PTB. In the multivariate regression analysis, the rs1800629 SNP also proved to be the only relevant predictor for PTB in mothers who were carriers of the GA or AA genotype. The rs1800896 SNP of the *IL-10* gene also proved to be a protective factor for early PTB and PTB in general for carriers of the G/G genotype in the recessive inheritance model. The rs1800869 SNP of the *IL-10* gene also increases the risk of PTB in mothers that are carriers of the GA heterozygote genotype in the superdominant inheritance model. Due to the limitations of our study, future studies in larger populations are needed to confirm our findings.

## Figures and Tables

**Table 1:** Single nucleotide polymorphisms (SNPs) of *IL-6*, *IL-10* and *TNFα* genes studied.

SNP	Location	Gene Region	Base Change	Citation	Related Phenotype
*TNFα* (rs1800629)	Chr 6:31575254 (GRCh38.p14)	Promoter region	G/A	Velez et al. [20] Yilmaz et al. [32] Jones et al. [36] Han et al. [30] Jie et al. [49] Drew.Piasecka et al. [50] Zhu et al. [48] Chen et al. [51]	Recurrent pregnancy loss Premature birth
*IL-10* (rs1800896)	chr 1:206773552 (GRCh38.p14)	Promoter region	T/C	Wagner et al. [52] Khorrami et al. [53] Čuljak et al. [54] Zhu et al. [47] Pandey et al. [26] Menon et al. [21] Lyubomirskaya et al. [25] Cao et al. [55]	Cervical cancer Alzheimer’s disease Breast cancer Preterm birth
*IL-6* (rs1800796)	Chr7: 22726627 (GRCh38.p14)	Promoter region	G/A	Shao et al. [56] Kaanene et al. [57] Santos et al. [58] Hou et al. [59] Luai et al. [60] Han et al. [30] Lyubomirskaya et al. [25]	Rheumatoid arthritis Lung cancer Gastritis and gastric cancer Chronic obstructive pulmonary disease Coronary artery disease Preterm birth

**Table 2:** Characteristics of mothers and infants born at term or premature.

	Term Birth (*n* = 200)	PTB (*n* = 199)	Total (*n* = 399)	*p*
Mother’s age (years) [Median (IQR)]	30 (26–34)	31 (27–35)		0.20 ^‡^
BMI (kg/m^2^) [Median (IQR)]	27.4 (24.5–30.6)	26.7 (24.2–30.1)		0.35 ^‡^
Underweight [*n* (%)]	2 (1)	0	2 (0.5)	0.15 ^†^
Normal weight [*n* (%)]	54 (27)	66 (33)	120 (30.1)	
Overweight [*n* (%)]	144 (72)	133 (67)	277 (69.4)	
Coffee consumption [*n* (%)]	167 (83.5)	155 (77.9)	322 (80.7)	0.17 *
Smoking habit [*n* (%)]	52 (25.9)	63 (31.5)	115 (28.7)	0.21 *
Pregnancy complications [*n* (%)]	104 (51.7)	150 (75.4)	254 (63.7)	<0.001 *
Uroinfection [*n* (%)]	18 (9)	27 (13.5)	45 (11.2)	0.15 *
Positive cervical swabs [*n* (%)]	44 (21.9)	35 (17.5)	79 (19.7)	0.27 *
Vaginal bleeding during pregnancy [*n* (%)]	14 (7)	45 (22.6)	59 (14.8)	<0.001 *
PPROM [*n* (%)]	47 (23.4)	118 (59.6)	165 (41.4)	<0.001 *
Number of previous births [Median (IQR)]	2 (1–2)	1 (1–2)		0.27 ^‡^
PB in family anamnesis [*n* (%)]	0	33 (16.5)	33 (8.2)	<0.001 *
Number of previous PB (*n* = 32) [Median (IQR)]	1 (*n* = 1)	1 (min 1–max 3)		**-**
Gestational age [Median (IQR)]	39 + 4 (39–40 + 3)	34 + 6 (32–36)		**<0.001 ^‡^**
Mode of delivery [*n* (%)]				
Vaginal birth	195 (98)	142 (71)	337 (84)	<0.001 *
Cesarean section	5 (2)	57 (29)	62 (16)	
Premature birth [*n* (%)]				
Extremely early PB	-	16 (8)	-	-
Early PB	-	32 (16)	-	-
Late PB	-	151 (76)	-	-
Birth weight of infants [g]	3450 (3123–3800)	2430 (1816–2780)		**<0.001 ^‡^**
Infant gender				
Male	100 (50)	117 (59)	217 (54)	0.07 *
Female	100 (50)	81 (41)	181 (46)	

* Chi-square test; ^†^ Fisher’s exact test; ^‡^ Mann–Whitney U test. Abbreviations: BM, body mass index; IQR, interquartile range; PPROM, preterm premature rupture of membranes, PB, premature birth. Bold denotes statistical significance.

**Table 3:** Genotype distribution and allele frequencies of three selected SNPs of genes of *TNFα, IL-6* and *IL-10* in mothers with PTB and respective controls in specific inheritance models.

SNP/Inheritance Models		Genotype [n (%)]		OR (95% CI)	p *
		Term Birth (*n* = 200)	PTB (*n* = 199)		
^a^ ***TNFα* (rs1800629)**	G/G	154 (77)	149 (74.9)	1	0.22
	A/G	44 (22)	43 (21.6)	1.01 (0.63–1.63)	
	A/A	2 (1)	7 (3.5)	3.62 (0.74–17.7)	
Alleles	G	352 (88)	341 (86)	0.82 (0.54–1.23)	0.3
	A	48 (12)	57 (14)		
Dominant inheritance model	G/G	154 (77)	149 (74.9)	1	0.62
	A/G–A/A	46 (23)	50 (25.1)	1.12 (0.71–1.78)	
Recessive inheritance model	G/G–A/G	198 (99)	192 (96.5)	1	0.08
	A/A	2 (1)	7 (3.5)	3.61 (0.74–17.59)	
Superdominant inheritance model	G/G–A/A	156 (78)	156 (78.4)	1	0.92
	A/G	44 (22)	43 (21.6)	0.98 (0.61–1.57)	
^b^ ***IL-10* (rs1800896)**	A/A	68 (34)	62 (31)	1	0.05
	A/G	88 (44)	109 (55)	1.36 (0.87–2.12)	
	G/G	44 (22)	28 (14)	0.70 (0.39–1.25)	
Alleles	A	224 (56)	233 (59)	1.11 (0.84–1.47)	0.47
	G	176 (44)	165 (41)		
Dominant inheritance model	A/A	68 (34)	62 (31.2)	1	0.54
	A/G–G/G	132 (66)	137 (68.8)	1.14 (0.75–1.73)	
Recessive inheritance model	A/A–A/G	156 (78)	171 (85.9)	1	**0.04**
	G/G	44 (22)	28 (14.1)	**0.58 (0.34–0.98)**	
Superdominant inheritance model	A/A–G/G	112 (56)	90 (45.2)	1	**0.03**
	A/G	88 (44)	109 (54.8)	**1.54 (1.04–2.29)**	
^c^ ***IL-6* (rs1800796)**	G/G	176 (88)	176 (88.4)	1	0.99
	C/G	23 (11.5)	22 (11.1)	0.96 (0.51–1.78)	
	C/C	1 (0.5)	1 (0.5)	1.00 (0.06–16.11)	
Alleles	G	375 (94)	374 (94)	1.04 (0.58–1.85)	0.90
	C	25 (6)	24 (6)		
Dominant inheritance model	G/G	176 (88)	176 (88.4)	1	0.89
	C/G–C/C	24 (12)	23 (11.6)	0.96 (0.52–1.76)	
Recessive inheritance model	G/G–C/G	199 (99.5)	198 (99.5)	1	>0.99
	C/C	1 (0.5)	1 (0.5)	1.01 (0.06–16.18)	
Superdominant inheritance model	G/G–C/C	177 (88.5)	177 (88.9)	1	0.89
	C/G	23 (11.5)	22 (11.1)	0.96 (0.51–1.78)	

^a^—Hardy–Weinberg equilibrium: PTB *p* = 0.14; term birth *p* = 0.75. ^b^—Hardy–Weinberg equilibrium: PTB *p* = 0.08; term birth *p* = 0.15. ^c^—Hardy–Weinberg equilibrium: PTB *p* = 0.52; term birth *p* = 0.55. * Chi-squared test. Abbreviations: SNP, single nucleotide polymorphism; PTB, premature birth; *IL-10*, interleukin 10; *IL-6*, interleukin 6; *TNFα*, tumor necrosis factor alpha; OR, odds ratio; CI, confidence interval. Bold denotes statistical significance.

**Table 4:** Genotype distribution of three selected SNPs of TNFα, IL-6 and IL-10 genes between three subgroups of PTB in mothers.

SNP		Genotype [n (%)]			*p* *
		Extremely Early PTB (*n* = 16)	Early PTB (*n* = 32)	Late PTB (*n* = 151)	
***TNFα* (rs1800629)**	A/A	0	0	7 (5)	0.91
	A/G	3 (19)	7 (22)	33 (22)	
	G/G	13 (81)	25 (78)	111 (73)	
***IL-10* (rs1800896)**	A/A	5 (31)	12 (38)	45 (30)	0.10
	A/G	6 (38)	19 (59)	84 (56)	
	G/G	5 (31)	1 (3)	22 (14)	
***IL-6* (rs1800796)**	C/C	0	0	1 (1)	0.89
	C/G	1 (6)	4 (13)	17 (11)	
	G/G	15 (94)	28 (88)	133 (88)	

* Fisher’s exact test. Abbreviations: SNP, single nucleotide polymorphism; PTB, premature birth; IL-10, interleukin 10; IL-6, interleukin 6; TNFα, tumor necrosis factor alpha; OR, odds ratio; CI, confidence interval. Bold denotes statistical significance.

**Table 5:** Genotype distribution and allele frequencies of three selected SNPs of genes of *TNFα, IL-6* and *IL-10* in mothers with extremely early PTB and respective controls in specific inheritance models.

SNP/Inheritance Model		Genotype [n (%)]		OR (95% CI)	p *
		Term Birth (*n* = 200)	Extremely Early PTB (*n* = 16)		
***TNFα* (rs1800629)**	G/G	154 (77)	13 (81.2)	1	0.81
	A/G	44 (22)	3 (18.8)	0.81 (0.22–2.96)	
	A/A	2 (1)	0	0 (0–NA)	
Allele	G	352 (88)	29 (91)	1.32 (0.39–4.49)	0.66
	A	48 (12)	3 (9)		
Dominant model of inheritance	G/G	154 (77)	13 (81.2)	1	0.69
	A/G–A/A	46 (23)	3 (18.8)	0.77 (0.21–2.83)	
Recessive model of inheritance	G/G–A/G	198 (99)	16 (100)	1	0.58
	A/A	2 (1)	0	0 (0–NA)	
Superdominant model of inheritance	G/G–A/A	156 (78)	13 (81.2)	1	0.76
	A/G	44 (22)	3 (18.8)	0.82 (0.22–3.0)	
***IL-10* (rs1800896)**	A/A	68 (34)	5 (31.2)	1	0.71
	A/G	88 (44)	6 (37.5)	0.93 (0.27–3.17)	
	G/G	44 (22)	5 (31.2)	1.55 (0.42–5.65)	
Allele	A	224 (56)	16 (50)	0.79 (0.38–1.62)	0.51
	G	176 (44)	16 (50)		
Dominant model of inheritance	A/A	68 (34)	5 (312)	1	0.82
	A/G–G/G	132 (66)	11 (68.8)	1.13 (0.38–3.39)	
Recessive model of inheritance	A/A–A/G	156 (78)	11 (68,8)	1	0.41
	G/G	44 (22)	5 (31.2)	1.61 (0.53–4.88)	
Superdominant model of inheritance	A/A–G/G	112 (56)	10 (62.5)	1	0.61
	A/G	88 (44)	6 (37.5)	0.76 (0.27–2.18)	
***IL-6* (rs1800796)**	G/G	176 (88)	15 (93.8)	1	0.73
	C/G	23 (11.5)	1 (6.2)	0.51 (0.06–4.04)	
	C/C	1 (0.5)	0	0 (0–NA)	
Allele	G	375 (94)	31 (97)	2,07 (0.27–15.77)	0.48
	C	25 (6)	1 (3)		
Dominant model of inheritance	G/G	176 (88)	15 (93.8)	1	0.46
	C/G–C/C	24 (12)	1 (6.2)	0.49 (0.06–3.87)	
Recessive model of inheritance	G/G–C/G	199 (99.5)	16 (100)	1	0.69
	C/C	1 (0.5)	0	0 (0–NA)	
Superdominant model of inheritance	G/G–C/C	177 (88.5)	15 (93.8)	1	0.44
	C/G	23 (11.5)	1 (6.2)	0.51 (0.06–4.07)	
	G/G	176 (88)	15 (93.8)	1	

* Chi-squared test. Abbreviations: SNP, single nucleotide polymorphism; PTB, premature birth; *IL-10*, interleukin 10; *IL-6*, interleukin 6; *TNFα*, tumor necrosis factor alpha; OR, odds ratio; CI, confidence interval. Bold denotes statistical significance.

**Table 6:** Genotype distribution and allele frequencies of three selected SNPs of genes of *TNFα*, *IL-6* and *IL-10* in mothers with early PTB and respective controls in specific inheritance models.

SNP/Inheritance Models		Genotype [*n* (%)]		OR (95% CI)	p *
		Term Birth (*n* = 200)	Early PTB (*n* = 32)		
***TNFα* (rs1800629)**	G/G	154 (77)	25 (78.1)	1	0.74
	A/G	44 (22)	7 (21.9)	0.98 (0.40–2.42)	
	A/A	2 (1)	0	0 (0–NA)	
Allele	G	352 (88)	57 (89)	1.11 (0.48–2.57)	0.81
	A	48 (12)	7 (11)		
Dominant inheritance model	G/G	154 (77)	25 (78.1)	1	0.89
	A/G–A/A	46 (23)	7 (21.9)	0-94 (0.38–2.31)	
Recessive inheritance model	G/G–A/G	198 (99)	32 (100)	1	0.44
	A/A	2 (1)	0	0 (0–NA)	
Superdominant inheritance model	G/G–A/A	156 (78)	25 (78.1)	1	0.99
	A/G	44 (22)	7 (21.9)	0.99 (0.40–2.45)	
***IL-10* (rs1800896)**	A/A	68 (34)	12 (37.5)	1	**0.01**
	A/G	88 (44)	19 (59.4)	1.22 (0.56–2.69)	
	G/G	44 (22)	1 (3,1)	0.13 (0.02–1.03)	
Allele	A	224 (56)	43 (67)	1.61 (0.92–2.81)	0.09
	G	176 (44)	21 (33)		
Dominant inheritance model	A/A	68 (34)	12 (37.5)	1	0.70
	A/G–G/G	132 (66)	20 (62.5)	0.86 (0.40–1.86)	
Recessive inheritance model	A/A–A/G	156 (78)	31 (96.9)	1	**0.003**
	G/G	44 (22)	1 (3.1)	**0.11 (0.02–0.86)**	
Superdominant inheritance model	A/A–G/G	112 (56)	13 (40.6)	1	0.11
	A/G	88 (44)	19 (59.4)	1.86 (0.87–3.97)	
***IL-6* (rs1800796)**	G/G	176 (88)	28 (87.5)	1	0.85
	C/G	23 (11.5)	4 (12.5)	1.09 (0.35–3.40)	
	C/C	1 (0.5)	0	0 (0–NA)	
Allele	G	375 (94)	60 (94)	1.0 (0.34–2.97)	>0.99
	C	25 (6)	4 (6)		
Dominant inheritance model	G/G	176 (88)	28 (87.5)	1	0.94
	C/G–C/C	24 (12)	4 (12.5)	1.05 (0.34–3.25)	
Recessive inheritance model	G/G–C/G	199 (99.5)	32 (100)	1	0.59
	C/C	1 (0.5)	0	0 (0–NA)	
Superdominant inheritance model	G/G–C/C	177 (88.5)	28 (87.5)	1	0.87
	C/G	23 (11.5)	4 (12.5)	1.10 (0.35–3.42)	

* Chi-squared test. Abbreviations: SNP, single nucleotide polymorphism; PTB, premature birth; IL-10, interleukin 10; IL-6, interleukin 6; TNFα, tumor necrosis factor alpha; OR, odds ratio; CI, confidence interval. Bold denotes statistical significance.

**Table 7:** Genotype distribution and allele frequencies of three selected SNPs of genes of *TNFα*, *IL-6* and *IL-10* in mothers with late premature birth and respective controls in specific inheritance models.

SNP/Inheritance Models		Genotype [n (%)]		OR (95% CI)	p *
		Term Birth (*n* = 200)	Late PTB (*n* = 151)		
***TNFα* (rs1800629)**	G/G	154 (77)	111 (73.5)	1	0.09
	A/G	44 (22)	33 (21.9)	1.04 (0.62–1.74)	
	A/A	2 (1)	7 (4.6)	4.86 (0.99–23.82)	
Allele	G	352 (88)	255 (84)	0.69 (0.45–1.07)	0.09
	A	48 (12)	47 (16)		
Dominant inheritance model	G/G	154 (77)	111 (73.5)	1	0.45
	A/G–A/A	46 (23)	40 (26.5)	1.21 (0.74–1.97)	
Recessive inheritance model	G/G–A/G	198 (99)	144 (95.4)	1	**0.03**
	A/A	2 (1)	7 (4.6)	4.81 (0.99–23.51)	
Superdominant inheritance model	G/G–A/A	156 (78)	118 (78.2)	1	0.97
	A/G	44 (22)	33 (21.9)	0.99 (0.59–1.65)	
***IL-10* (rs1800896)**	A/A	68 (34)	45 (29.8)	1	0.07
	A/G	88 (44)	84 (55.6)	1.44 (0.89–2.33)	
	G/G	44 (22)	22 (14.6)	0.76 (0.40–1.43)	
Allele	A	224 (56)	174 (58)	1.07 (0.79–1.44)	0.67
	G	176 (44)	128 (42)		
Dominant inheritance model	A/A	68 (34)	45 (29.8)	1	0.40
	A/G–G/G	132 (66)	106 (70.2)	1.21 (0.77–1.91)	
Recessive inheritance model	A/A–A/G	156 (78)	129 (85.4)	1	0.08
	G/G	44 (22)	22 (14.6)	0.60 (0.34–1.06)	
Superdominant inheritance model	A/A–G/G	112 (56)	67 (44.4)	1	**0.03**
	A/G	88 (44)	84 (55.6)	**1.60 (1.04–2.44)**	
***IL-6* (rs1800796)**	G/G	176 (88)	133 (88.1)	1	0.98
	C/G	23 (11.5)	17 (11.3)	0.98 (0.50–1.90)	
	C/C	1 (0.5)	1 (0.7)	1.32 (0.08–21.35)	
Allele	G	375 (94)	283 (94)	0.99 (0.54–1.84)	0.98
	C	25 (6)	19 (6)		
Dominant inheritance model	G/G	176 (88)	133 (88.1)	1	0.98
	C/G–C/C	24 (12)	18 (11.9)	0.99 (0.52–1.90)	
Recessive inheritance model	G/G–C/G	199 (99.5)	150 (99.3)	1	0.84
	C/C	1 (0.5)	1 (0.7)	1.33 (0.08–21.38)	
Superdominant inheritance model	G/G–C/C	177 (88.5)	134 (88.7)	1	0.94
	C/G	23 (11.5)	17 (11.3)	0.98 (0.50–1.90)	

* Chi-squared test. Abbreviations: SNP, single nucleotide polymorphism; PTB, premature birth; IL-10, interleukin 10; IL-6, interleukin 6; TNFα, tumor necrosis factor alpha; OR, odds ratio; CI, confidence interval. Bold denotes statistical significance.

**Table 8:** Frequency of haplotypes of three SNPs and their possible association with PTB in probands.

Haplotype			n (%)		OR (95%CI)	χ^2^	*p* *	_adj_*p* ^†^
			PTB	Term Birth				
Mothers			χ^2^ = 2.41 df = 4 *p* = 0.66					
rs1800629	rs1800896	rs1800796						
G	G	G	117 (29.3)	134 (33.5)	0.83 (0.61–1.11)	1.56	0.22	0.42
G	A	G	210 (52.7)	199 (49.7)	1.13 (0.85–1.49)	0.73	0.21	0.55
A	G	G	29 (7.2)	25 (6.2)	1.18 (0.68–2.05)	0.34	0.56	0.64
A	A	G	18 (4.5)	17 (4.2)	1.07 (0.54–2.10)	0.67	0.41	0.85
G	G	C	10 (2.5)	14 (3.5)	0.71 (0.31–1.62)	0.04	0.85	0.85

Abbreviations: PTB, premature birth; OR odds ratio; CI, confidence interval. Bold denotes statistical significance. ^†^ Correction of *p* value by Benjamini–Hochberg method. * Chi-squared test.

**Table 9:** Prediction of probability for PTB (bivariant and multivariant (backward) regression analysis).

	ß	Wald	*p*	OR (95 CI%)
* Bivariant logistic regression				
*TNFα* (rs1800629) (A/G–A/A vs. G/G)	0.738	5.47	**0.02**	2.09 (1.13–3.88)
*IL-10* (rs1800896) (A/G–G/G vs. A/A)	0.105	0.22	0.64	1.11 (0.72–1.71)
*IL-6* (rs1800796) (C/G–C/C vs. G/G)	−0.225	0.46	0.50	0.80 (0.42–1.53)
* Multivariant logistic regression				
*TNFα* (rs1800629) (A/G–A/A vs. G/G)	0.74	5.47	**0.02**	2.10 (1.13 to 3.88)
*Constant*	−0.76	0.66	**0.04**	

Abbreviations: ß—regression coefficient; OR—odds ratio; CI—confidence interval; *TNFα*, tumor necrosis factor alpha; *IL-10*, interleukin 10; *IL-6*, interleukin 6. * adjusted for smoking habit, mothers age, body mass index, newborns gender and vaginal bleeding during pregnancy. Bold denotes statistical significance.

## Data Availability

The datasets generated and analyzed during the current study are not publicly available due to the informed consent given by patients in this study which does not cover data posting in public databases. However, data are available upon reasonable request and requests should be sent to mirta.kadivnik@gmail.com.
